# Effect of sedatives or anesthetics on the measurement of resting brain function in common marmosets

**DOI:** 10.1093/cercor/bhac406

**Published:** 2022-10-12

**Authors:** Kanako Muta, Junichi Hata, Naoki Kawaguchi, Yawara Haga, Daisuke Yoshimaru, Kei Hagiya, Takaaki Kaneko, Takako Miyabe-Nishiwaki, Yuji Komaki, Fumiko Seki, Hirotaka James Okano, Hideyuki Okano

**Affiliations:** Graduate School of Human Health Sciences, Tokyo Metropolitan University, Arakawa, Tokyo 116-8551, Japan; Division of Regenerative Medicine, The Jikei University School of Medicine, Minato, Tokyo 105-8461, Japan; Graduate School of Human Health Sciences, Tokyo Metropolitan University, Arakawa, Tokyo 116-8551, Japan; Division of Regenerative Medicine, The Jikei University School of Medicine, Minato, Tokyo 105-8461, Japan; Laboratory for Marmoset Neural Architecture, Center for Brain Science, RIKEN, Wako, Saitama 351-0198, Japan; Department of Physiology, Keio University School of Medicine, Shinjuku, Tokyo 160-8582, Japan; Graduate School of Human Health Sciences, Tokyo Metropolitan University, Arakawa, Tokyo 116-8551, Japan; Graduate School of Human Health Sciences, Tokyo Metropolitan University, Arakawa, Tokyo 116-8551, Japan; Laboratory for Marmoset Neural Architecture, Center for Brain Science, RIKEN, Wako, Saitama 351-0198, Japan; Live Imaging Center, Central Institute for Experimental Animals, Kawasaki, Kanagawa 210-0821, Japan; Division of Regenerative Medicine, The Jikei University School of Medicine, Minato, Tokyo 105-8461, Japan; Laboratory for Marmoset Neural Architecture, Center for Brain Science, RIKEN, Wako, Saitama 351-0198, Japan; Department of Physiology, Keio University School of Medicine, Shinjuku, Tokyo 160-8582, Japan; Live Imaging Center, Central Institute for Experimental Animals, Kawasaki, Kanagawa 210-0821, Japan; Laboratory for Marmoset Neural Architecture, Center for Brain Science, RIKEN, Wako, Saitama 351-0198, Japan; Laboratory for Marmoset Neural Architecture, Center for Brain Science, RIKEN, Wako, Saitama 351-0198, Japan; Systems Neuroscience Section, Primate Research Institute, Kyoto University, Inuyama, Aichi 484-8506, Japan; Center for Model Human Evolution Research, Primate Research Institute, Kyoto University, Inuyama, Aichi 484-8506, Japan; Department of Physiology, Keio University School of Medicine, Shinjuku, Tokyo 160-8582, Japan; Live Imaging Center, Central Institute for Experimental Animals, Kawasaki, Kanagawa 210-0821, Japan; Department of Physiology, Keio University School of Medicine, Shinjuku, Tokyo 160-8582, Japan; Live Imaging Center, Central Institute for Experimental Animals, Kawasaki, Kanagawa 210-0821, Japan; Division of Regenerative Medicine, The Jikei University School of Medicine, Minato, Tokyo 105-8461, Japan; Laboratory for Marmoset Neural Architecture, Center for Brain Science, RIKEN, Wako, Saitama 351-0198, Japan; Department of Physiology, Keio University School of Medicine, Shinjuku, Tokyo 160-8582, Japan

**Keywords:** anesthetic effects, common marmoset, resting-state functional magnetic resonance imaging, resting state network

## Abstract

Common marmosets are promising laboratory animals for the study of higher brain functions. Although there are many opportunities to use sedatives and anesthetics in resting brain function measurements in marmosets, their effects on the resting-state network remain unclear. In this study, the effects of sedatives or anesthetics such as midazolam, dexmedetomidine, co-administration of isoflurane and dexmedetomidine, propofol, alfaxalone, isoflurane, and sevoflurane on the resting brain function in common marmosets were evaluated using independent component analysis, dual regression analysis, and graph-theoretic analysis; and the sedatives or anesthetics suitable for the evaluation of resting brain function were investigated. The results show that network preservation tendency under light sedative with midazolam and dexmedetomidine is similar regardless of the type of target receptor. Moreover, alfaxalone, isoflurane, and sevoflurane have similar effects on resting state brain function, but only propofol exhibits different tendencies, as resting brain function is more preserved than it is following the administration of the other anesthetics. Co-administration of isoflurane and dexmedetomidine shows middle effect between sedatives and anesthetics.

## Introduction

The common marmoset (*Callithrix jacchus*), a promising laboratory animal, is a species of new-world monkeys classified in the genus *Callithrix* in the marmoset family native to South America. They have many well-established characteristics as model animals, which are not only advantageous for breeding—such as small body size, 20–30 cm height, 350–400 g body weight, and calm character (Okano et al. 2012)—but also advantageous in a neural structure compared with rodents having similar body size. Despite their body size, they have a highly developed cerebral cortex and retain a structure unique to primates. These features are major advantages in translational research in higher-order brain functional research; therefore, marmosets are in demand as preclinical models in higher brain function research (Okano 2020). Furthermore, they have high reproductive efficiency, early sexual maturation (at approximately 1.5 years old), relatively large litter sizes (2–3 pups per delivery), and relatively short gestation periods and delivery intervals (145–148 and 154–157 days, respectively) (Okano 2020); and these features are suitable for genetic modification and systematization. Determination of the whole genome sequence (Worley et al. 2014), creation of transgenic marmosets of polyglutamine disease models using genetic modification techniques (Tomioka et al. 2017), and systematization of transgenic marmosets (Sasaki et al. 2009) have already been reported. A project for the creation and systematization of disease models using these techniques in marmosets is ongoing (Okano and Mitra 2015; Okano et al. 2016). As mentioned above, these factors represent substantial advantages as a preclinical model and are unique to marmosets. Researching brain function through these transgenic model marmosets would help us in overcoming diseases causing higher-order brain function disorder and eventually advance the investigation of human brain function (Feng et al. 2020; Okano 2020).

Computed tomography, positron emission tomography, and magnetic resonance imaging are widely used as in vivo neural activity visualization techniques in human brain research. Because the results obtained using these techniques can be easily applied to clinical situations as translational research, research reports using these techniques in marmosets have recently increased (Belcher et al. 2013; Liu et al. 2013, 2019; Hori et al. 2019); and it is expected that these will also become the primary techniques for higher-order brain function research in marmosets. Resting-state functional magnetic resonance imaging (rs-fMRI) is a method used in brain mapping to evaluate regional interactions that occur in a resting or task-negative state based on spontaneous fluctuations in blood oxygen level-dependent signal. These regional interactions are named “resting-state networks (RSNs),” and these RSNs can be classified into higher-order cognitive network and lower-order sensory network. Default mode network, a kind of higher-order cognitive network, has been reported to change in higher-order brain function disorders such as Alzheimer’s disease (Sorg et al. 2007; Agosta et al. 2012) and autism (Cherkassky et al. 2006; Weng et al. 2010). This indicates that higher-order cognitive networks relate to higher-order brain function (Raichle 2010, 2011, 2013); therefore, investigating RSNs, especially higher-order cognitive network in marmosets as a promising preclinical animal model would be beneficial for higher-order brain function research.

One of the main problems is immobilizing animals in laboratory fMRI studies. fMRI is performed under general anesthesia in many cases, but anesthetics affect brain function. (Peltier et al. 2005; Boveroux et al. 2010). It is possible to measure marmosets’ brain function without any sedates/anesthetics but a head fixation with a fixing instrument (Hutchison et al. 2012; Gorges et al. 2017); however, not all institutes use similar fixtures. Many fMRI investigations employ anesthetics to mitigate the effects of movement, physiological stress, and training demands (Consortium et al. 2020). Understanding anesthetics’ effects on RSNs by comparing RSNs under various anesthetic conditions and exploring appropriate anesthetic maintenance methods is important to promote marmoset-based higher-order brain functional research.

Anesthetics induce sedation or hypnotic effects by binding or affecting various receptors expressed on neurons and mediate cells to inhibit the action potential of neurons. Differences in these working mechanisms, target receptors, and pharmacokinetics appear as differences in anesthetic or sedative effects. Research comparing RSNs under awake and 6 different anesthetic conditions in rats shows that various anesthetics affect RSNs differently (Paasonen et al. 2018). In this report, isoflurane (Iso), commonly used as a general anesthetic agent in fMRI marmoset research, greatly changed brain connectivity while propofol (Propo), commonly used in human and veterinary clinical settings, had the least effect on it. It can be expected that similar effects on marmoset RSNs will occur. Some studies to investigate the effect of anesthetics on the brain function in marmosets have already been reported. Isoflurane is useful to evaluate the global structure of functional networks but may obfuscate important nodes of some network components when compared to data acquired in fully awake marmosets (Hori et al. 2020). Liu et al. reported that propofol significantly influenced the shape of the hemodynamic response function and the interareal spontaneous functional connectivity (Liu et al. 2013). However, there are no reports investigating the effect of other sedatives/anesthetics.

The purpose of this study is to investigate the most suitable anesthetic or sedative for higher-order brain function in marmosets among midazolam (Mida), dexmedetomidine (Dex), Propo, alfaxalone (Alfa), Iso, sevoflurane (Sevo), and co-administration of Iso and Dex (IsoDex), which are commonly used in human and veterinary clinical settings. Independent component analysis (ICA) and graph theoretical analysis with betweenness centrality, which indicates that the region with high betweenness centrality is considered as the most crucial region for efficient information transfer between local clusters, were performed to detect RSNs and evaluate which RSN-constructing region is particularly affected by sedatives/anesthetics and leads to the loss of the RSNs in this study. Furthermore, in higher-order cognitive network, a more detailed analysis with dual regression analysis was carried out to quantify the effect inhibiting synchronization between brain regions to understand the effects of sedatives/anesthetics for investigating higher-order brain function.

The hypothesis was as follows. Mida: all RSNs will be detected with ICA but betweenness centrality in regions constructing higher-order cognitive network will be decreased. Mida is a GABA receptor agonist, and GABA receptors are expressed throughout the cerebral cortex. Although higher-order cognitive network and lower-order sensory network were also detected under sedation with Mida, resting-state functional connectivity among brain regions constructing higher-order cognitive network was inhibited and activated among brain regions constructing lower-order sensory network in humans (Liang et al. 2015). Similar phenomena are expected to be observed under Mida condition in marmosets. Dex: all RSNs will be detected with ICA but betweenness centrality in regions constructing higher-order cognitive network will be decreased. However, Dex is expected to have a smaller effect on the RSNs than that of Mida because Dex is an agonist for the alpha2 receptor expressed on the axonal endings of noradrenergic neurons, and the target neurons are more limited than the GABA receptor agonists are. Propo, Alfa, Iso, and Sevo: they are expected to have a greater effect, more RSNs become undetectable and betweenness centrality in many regions constructing not only higher-cognitive networks but also lower-order sensory networks, is decreased compared to Mida and Dex. They are classified as anesthetics and are known to act on various receptors. In addition to this, based on previous reports, this study was conducted based on the assumption that co-administration of IsoDex would have the least effect on RSNs.

## Materials and methods

### Animals

This study was approved by the Animal Experiment Committees at the RIKEN Center for Brain Science (CBS) and was conducted per the guidelines for Conducting Animal Experiments of RIKEN CBS. Three male and one female healthy common marmosets (*C. jacchus*) between 3 and 6 years of age were included. The marmosets were housed in a room where temperature and humidity were maintained at 26–30 °C and 30%–70%, respectively, with a 12-h light–dark cycle. Water was provided ad libitum. They were fed 40 g pellets supplied with vitamins and minerals once a day. All marmosets were not fed in the morning before the experiments, but water was freely available until immediately before and after the experiment. A highly palatable food was fed as a reward after imaging, when they completely recovered from sedation/anesthesia except for awake condition. All marmosets were examined 8 times to collect functional MRI data in all conditions. After awake data were firstly collected, and sedate/anesthetic data were done in a random order for sedate/anesthetic condition with an interval of 1 month between each examination in each individual.

### Headpost insertion surgery

All marmosets underwent a surgical procedure to attach a headpost to the cranial bones to prevent head movement during data collection. They were not fed at night before surgery, but water was freely available. They were carried into the operating room in a carrying cage on the day of the operation. 1 mg/head (2.1–2.9 mg/kg) of ketamine and 10 μg/head (20.8–28.5 μg/kg) of medetomidine were administered intramuscularly as a premedication, and 3% Iso (MSD Animal Health Japan, Tokyo, Japan) with 100% O_2_ was administered as a carrier gas through a facial mask when the escape behavior for wearing the face mask disappeared after premedication.

When muscular relaxation was observed, local anesthesia of the larynx was performed with lidocaine hydrochloride jelly (Xylocine Jelly 2%, Aspen Japan, Tokyo, Japan), and an 8 Fr catheter (Atom multi-use tube, Atom Medical Corp., Tokyo, Japan) was inserted as an intratracheal tube. After intubation, the intratracheal tube was connected to an artificial ventilator (SN-480-7, Shinano Seisakusho, Tokyo, Japan), and mechanical ventilation was performed with a 50% concentration of intake oxygen, 8 mL of tidal volume, and 30 respiratory rate (RR) per min.

General anesthesia was maintained with 2.0% Iso after intubation, and pulse rate (PR), percutaneous arterial blood oxygen saturation (SpO_2_), RR, end-tidal carbon dioxide concentration (EtCO_2_), intake and expired Iso concentration, and rectal temperature were monitored using a vital sign monitor (Quebec, Canada, Bedside SpO_2_ Patient Monitoring System, COVIDIEN, Dublin, Ireland, AccuSens fiber optic sensor, Opsens, BP-608 Evolution, Omron-Collin, Tokyo, Japan). After monitoring was stable, the marmoset heads were fixed with Stereotaxic Instruments for Common Marmosets (SR-6C-HT, Narishige Group, Tokyo, Japan), and hair from the crown of the head was shaved. 7.2 mg/head of Cefovecin sodium (Convenia, Zoetis Japan, Tokyo, Japan) to prevent surgical site infection, 0.3 mg/kg of meloxicam (Metacam, Boehringer Ingelheim Japan, Osaka, Japan) for analgesia, and 5 mL/kg of lactated ringer fluid to prevent dehydration were administrated subcutaneously. A heat mat was placed under the marmosets to prevent hypothermia.

After disinfection of the skin of the crown of the head with Isodine, marmosets were covered with a drape, and the surgical site was covered with an antimicrobial incise drape (Ioban, 3 M Japan, Tokyo, Japan). The skin of the cranial head was incised from the center of the forehead to the back of the head, and the periosteum and temporal muscles were separated from the cranial bones and Linea temporalis, respectively. The insertion location of the head post was determined using a model of a receiver coil manufactured for the common marmoset, and the headpost was bonded vertically using dental cement (Super-Bond C&B Kit, Sun Medical, Shiga, Japan). After the cement setting, the skin was sutured with a 5-0 nylon thread, and Iso administration was terminated. When the deglutition reflex was observed, the intratracheal tube was extubated, and the marmosets were treated in an intensive care unit for animals. For postoperative analgesia, 0.05 mg/kg of meloxicam was administered orally from the day after surgery for three days.

### Anesthetic or sedative management for data collection

Because anesthetic agents affect brain activities depending on anesthetic depth (Boveroux et al. 2010; Grandjean et al. 2014), a minimal dose that enables immobilization for non-painful stimulus in Mida and Dex or intratracheal intubation in Propo, Alfa, Iso, and Sevo was administrated. Immobilized condition in Mida and Dex was defined as “no response to sound stimulation but eyes are opened when the body is rocking. (This condition is equivalent to sedation score 3 for both response to sound stimulation and to direct stimulation in the primate sedation score reported by Miyabe et al. (2001) and Muta et al. (2021).” All doses except for IsoDex were decided based on a preliminary study. The doses of IsoDex were determined based on a previous study reported by Hayashi et al. (2021). It was confirmed in a preliminary study that this dose enables prevention of spontaneous movement against sound and direct stimulation (sedation score of response to sound stimulation is 3 and to direct stimulation is 4 in the primate sedation score), but not against intubation to an endotracheal tube.

#### Midazolam

A marmoset was restrained with leather gloves, and an in-dwelling needle (Safelet Cath 24G 3/4″, NIPRO, Osaka, Japan) was inserted into the tail vein. Subsequently, 0.25 mg/kg of Mida (Dormicum Injection, Maruishi Pharmaceutical. Co. Ltd, Osaka, Japan) was administrated over 1 min for induction, and continuous rate infusion was performed at the rate of 0.6 mg/kg/h with a microsyringe pump. These dose and administration rates were determined based on a previous study (Uehara et al. 2015) and a preliminary study. In the preliminary study, intratracheal intubation and artificial ventilation were not performed during sedation with Mida, and 50% O_2_ was administered through a facial mask. SpO_2_, PR, and RR were monitored, but other vital signs, rectal temperature, and EtCO_2_ were hard to measure because only light sedation was obtained, and setting a rectal temperature monitor and a gas sampling tube was difficult.

#### Dexmedetomidine

The setting of an in-dwelling needle was performed in the same way as for Mida. Dex (10 μg/kg; Precedex, Pfizer Japan Inc., Tokyo, Japan) was administered at a rate of 5 μg/kg/min, and continuous rate infusion was performed at a rate of 20–40 μg/kg/h. The dose and infusion rate were determined based on a previous report in rats (Magnuson et al. 2014) and a preliminary study. O_2_ administration and vital signs monitoring were managed in the same way as for Mida because sufficient sedation to intubate the trachea could not be obtained with Dex as with Mida.

#### Co-administration of isoflurane and dexmedetomidine

Marmosets were induced with a face mask in the same way as for Iso. After reaching sufficient sedation for setting an in-dwelling needle, Iso concentration for administration was reduced to 2.5%, and an in-dwelling needle was inserted into the tail vein. 2.5 μg/kg of Dex was administered via the in-dwelling needle with a microsyringe pump over 2 min as a loading dose, and Iso concentration was reduced to 0.5% at the same time. Immediately after administrating the loading dose, continuous rate infusion was performed at a rate of 5 μg/kg/h. RR was managed, and vital signs were monitored in the same way as for Mida because sufficient sedation to intubate the trachea could not be obtained with this protocol as for Mida.

#### Propofol

An in-dwelling needle was set in the same way as for Mida. Propo (12 mg/kg) was administered as induction of general anesthesia over 3 min via the in-dwelling needle. Atropine (50 μg/kg) was administered subcutaneously to prevent intratracheal secretion. Immediately after bolus administration, the predicted plasma concentration of Propo was titrated to 7–9 μg/mL for intratracheal intubation with continuous infusion. The administration dose and protocol were calculated beforehand using a pharmacokinetic parameter reported by Muta et al. (2021) and pharmacokinetic analysis software, NONMEM ver. VII (GloboMax ICON Development Solutions, Ellicott City, MD, USA). Once a sufficient plasma concentration was obtained, the Propo dose was controlled to maintain that concentration, and an 8 Fr catheter (Atom multi-use tube, Atom Medical Corp., Tokyo, Japan) was inserted into the trachea as an intratracheal tube.

After intratracheal intubation, the marmosets were connected to artificial ventilation for small animals (SN-480-7, Shinano Seisakusho, Tokyo, Japan), and mechanical ventilation was performed under the following conditions: 50% inspiratory oxygen, 8 mL of tidal volume, and 30 breaths per min RR. PR, SpO2, RR, EtCO2, inspiratory and end-tidal Iso concentrations, and rectal temperature were measured with a vital sign monitor.

#### Alfaxalone

The setting of an in-dwelling needle, induction of general anesthesia, and administration of atropine were carried out in the same way as for Propo. Alfaxalone (5 mg/kg) was administered over 2 min for induction, and general anesthesia was maintained with a continuous infusion at 20–25 mg/kg/h. Intratracheal intubation, respiratory management, and vital sign monitoring were performed in the same manner as that for Propo. The dose and administration protocol were decided through a preliminary because the pharmacokinetic parameters have not been reported, unlike Propo.

#### Isoflurane

Three percent Iso with 100% O_2_ as carrier gas was administered through a facial mask to the marmosets retained with leather gloves. Once sufficient sedation was achieved, the Iso concentration was reduced to 2.5%, and 50 μg/kg of atropine and 3.0 mL of physiological saline were administered subcutaneously to prevent intratracheal secretion and dehydration, respectively. Intratracheal intubation, respiratory management, and vital sign monitoring were performed in the same manner as for Propo. After intratracheal intubation, general anesthesia was maintained with 1.8% Iso.

#### Sevoflurane

All procedures were performed in the same way as Iso, except for dosages. The induction of general anesthesia, intratracheal intubation, and maintenance of general anesthesia were performed with 5.0 and 3.0% of Sevo (Sevoflurane, Pfizer Japan Inc., Tokyo, Japan), respectively.

### Data collection condition

During imaging, the marmosets were placed on a custom-made imaging table (Takashima Seisakusho Co., Ltd, Tokyo, Japan) and immobilized by fixing the head post using a head post fixing tool attached at a custom-made imaging table in all conditions. The marmosets were fitted with earplugs. A hot water circulator was used during imaging to maintain body temperature 36–38 °C under all conditions.

In Awake condition, data were collected in the dark and monitored with an infrared camera to prevent the marmosets from falling asleep. If the marmosets were observed closing their eyes during a scan, they were awakened with a loud noise before the next scan was started. They were rewarded with a highly palatable food at the end of each imaging.

In sedate/anesthetic condition, the marmosets were monitored in the same way to observe spontaneous movement or coughing for intratracheal tube. If spontaneous movement or coughing was observed, additional sedatives/anesthetics were administrated and infusion rate or concentration of inhalational anesthesia was increased.

### Data acquisition

An ultra-high field MRI system with a static magnetic field strength of 9.4 T (Bruker BioSpin, Ettlingen, Germany), a custom-made 8-channel receiver coil for the marmoset head (Takashima Seisakusho Co., Ltd, Tokyo, Japan), and a 154 mm inner diameter transmitter coil (Bruker BioSpin, Ettlingen, Germany) were used to collect structural and functional data. Structural data and T2-weighted images were imaged using rapid acquisition with relaxation enhancement (RARE) sequence with the following conditions and parameters: time repetition (TR) = 4,331 ms, time echo (TE) = 15.0 ms, FOV = 42.0 × 28.0 × 36.0 mm, matrix size = 120 × 80 voxels, resolution = 0.35 × 0.35 mm, slice thickness = 0.7 mm, number of slices = 52, scan time = 1 min and 26 s, RARE factor = 4. Functional images were captured using a gradient recalled echo-planar imaging (EPI) sequence with the following conditions and parameters: TR = 2,000 ms, TE = 16.0 mm, FOV = 42.0 × 28.0 × 36.0,mm matrix size = 60 × 40 voxels, resolution = 0.7 × 0.7 mm, slice thickness = 0.7 mm, number of slices = 52, repetition = 155, scan time = 5 min and 10 s. Functional imaging was performed 12 times per subject. These data were treated as 60 min data from each individual.

### Preprocessing

After the acquired data were converted to Neuro Informatics Technology Initiative format (NIfTI), the voxel size was changed from 0.7 mm isotropic to 3.5 mm isotropic using SPM (Wellcome Trust Center for Neuroimaging, London, UK). Estimation and correction of geometric distortions induced by magnetic susceptibility were performed with the top-up tool of the FMRIB Software Library (FSL) software (FMRIB, Oxford, UK) because all cross-sections were imaged with a single excitation in EPI. Slice timing correction was performed to correct for signal acquisition timing discrepancies in each section. Realignment was applied to compensate for head movements caused by body movements. The deviations in 6 directions were obtained: *x* (left/right), *y* (front/back), *z* (up/down), pitch (rotational direction of nodding and looking up), roll (rotational direction of moving the ear closer to the shoulder), and yaw (rotational direction of looking left/right). For each measurement time point (TR), the deviation from the reference time point, and the first functional brain image, was determined; and the image was moved and rotated by the rigid body model based on this deviation. The method of finding the parameters of the linear transformation was used to minimize the difference between the first functional brain image and the affine transformation of the series of functional brain images to be corrected, by calculating convergence using the method of least squares. After correcting the spatial scale error between the structural and functional images with co-registration, segmentation was performed to provide information on the tissue to which each voxel belongs in terms of brain tissue classification. The voxels were spatially standardized by normalization, which aligns the voxels to the standard brain image to correct for structural differences between individuals. Smoothing was applied to suppress excessive voxel value fluctuations within individuals and apply normal probability field theory. Functional data were smoothed using spatial convolution with a Gaussian kernel of 2 voxels (7 mm). Then, physiological noise was denoised using ordinary least squares regression with cerebrospinal fluid pulsation, heart rate, and respiratory artifacts as regressors. Temporal band pass filtering was performed by frequency filtering (0.01–0.1 Hz) using the fMRI denoising pipeline of CONN (Gabrieli Lab. McGovern Institute for Brain Research, MIT, USA). In this study, brain region data were divided into 52 and 104 regions in the cerebral cortex of the hemisphere and whole brain, respectively (Table 2), based on the common marmoset atlas data reported by Hashikawa et al. (2015).

### Independent component analysis

Probabilistic ICA was performed in each condition to detect RSNs using multivariate exploratory linear optimized decomposition into the independent components (MELODIC) module of the FSL software package (Beckmann 2012). Preprocessed and denoised data were whitened and projected into a 25-dimensional subspace using principal component analysis. This subspace was chosen to be a suitable representative of significant components by referring to prior studies of marmoset functional networks (Belcher et al. 2013; Ghahremani et al. 2016; Hori et al. 2020). The ICA analysis was implemented 5 times with varied dimension numbers (15, 20, 25, 30, and 35) to discover optimal dimensionality. By applying a fixed-point iteration technique to optimize for non-Gaussian spatial source distributions, the whitened observations were divided into sets of vectors that characterize signal change throughout the temporal domain (timecourses) and across the spatial domain (maps). By fitting a mixture model to the histogram of intensity values, the estimated component maps were split by the residual noise standard deviation and thresholded (*z*-value). RSNs were defined as components in which voxels with *z*-values of 2.5% or higher were detected approximately symmetrically and did not contain white matter, medulla oblongata, large blood vessels, or ventricles. Voxels with *z*-values of 2.5% or higher were extracted from the ICA results and the regions where voxels were more than 5% of the total number of voxels constituted. When these regions were detected in both hemispheres, they were determined as regions constituting RSNs.

### Dual regression analysis

To compare RSNs in each sedate/anesthetic condition with the awake condition, the voxels whose timecourse was statistically changed with administration of sedatives/anesthetics were detected in each RSN with the dual regression module of the FSL software package. In this analysis, the set of spatial maps from the group-average analysis was used to generate subject-specific versions of the spatial maps and associated time series using dual regression (Nickerson et al. 2017).

First, the whole-group ICA, which was apart from the aforementioned group-ICA, was performed with all preprocessed data from all conditions to identify large-scale special patterns to be applied as spatial templates and the group-average set of spatial maps was regressed (as spatial regressors in a multiple regression) into each subject’s four-dimensional (4D) space–time dataset. The 25-component solution was chosen as with the above-mentioned group—ICA. This results in a set of subject-specific time series, one per group-level spatial map. Next, those time series were regressed (as temporal regressors, again in a multiple regression) into the same 4D dataset, resulting in a set of subject-specific spatial maps, one per group-level spatial map. We then tested for group differences using the FSL package’s random permutation testing tool with 5,000 permutations. In permutation testing, threshold-free cluster enhancement was adopted for finding clusters, and then multiple comparisons were performed among these clusters. The probability value was corrected using Bonferroni correction. Statistical significance was considered when the probability value was less than 0.05/(number of components used in the comparison × 2). In this study, *P* < 0.002 was considered statistically significant.

### Graph theoretic analysis

Partial correlation coefficients were calculated instead of correlation coefficients to eliminate the effect of other continuous variables between regions for the evaluation of functional connectivity. Partial correlation coefficients were obtained from the preprocessed time-series data of each individual using ridge regression. The difference in partial correlation coefficients was calculated to evaluate their change between the awake and sedated/anesthetized conditions. If the partial correlation coefficients in the awake and sedated/anesthetized states had the same sign (positive or negative), the difference in absolute value was calculated by subtracting the absolute value of partial correlation coefficients of sedated/anesthetized condition from the awake condition. If the partial correlate coefficients changed across 0 due to the administration of sedatives/anesthetics, the amount of increase or decrease was indicated instead of the difference in absolute values.

To evaluate each region’s information transfer function, the betweenness centralities among 52 regions in each hemisphere were calculated by binarizing the partial correlate coefficients at a threshold set to 0.1, which is: mean + standard deviation × 2 of awake condition of the partial correlation coefficients. If the partial correlation coefficients were larger than 0.1, functional connectivity was assumed between regions. For the default mode network (DMN) and dorsal attention network (DAN), betweenness centralities were calculated among the regions detected as constituent regions by ICA. Statistical analysis was performed to compare betweenness centrality between the awake condition and other sedate/anesthetic conditions. Betweenness centrality calculated among 52 regions in each hemisphere was treated as the data obtained from 8 hemispheres. One-way analysis of variance (ANOVA) was firstly performed and Tukey–Kramer test was carried out in regions found with significant differences in one-way ANOVA. *P* < 0.05 was considered as significant in all statistical analyses. MATLAB (The MathWorks, Natick, USA) and R software (Ihaka and Gentleman 1996) (http://www.r-project.org/) were used to calculate the partial correlate coefficients, betweenness centralities, and statistical analyses.

## Results

### RSNs detected in each condition

**Fig. 1:**
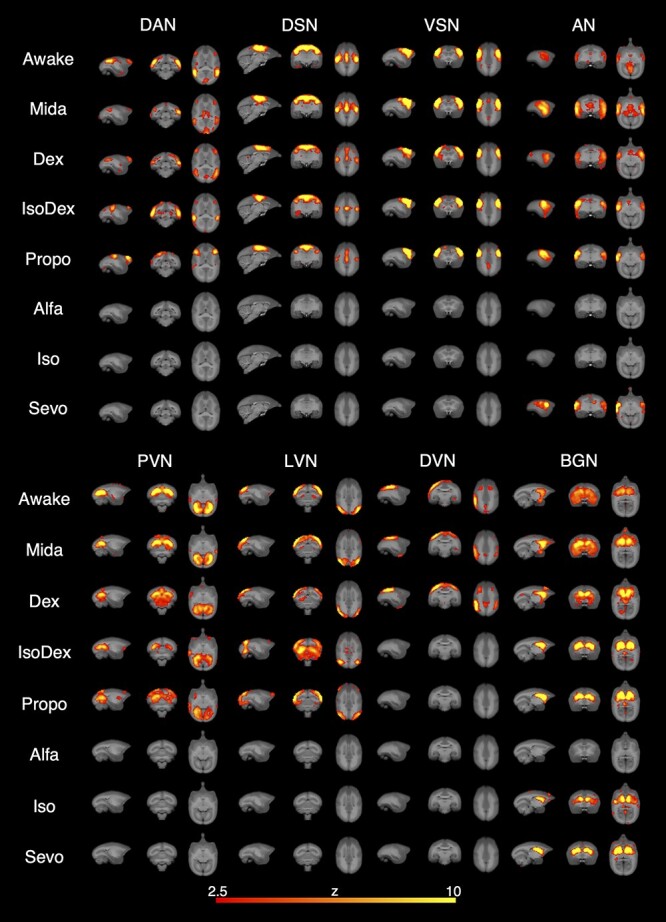
RSNs detected in each condition resulting group-ICA. Voxels with *z*-scores >2.5 are labeled. The 3 brain images in each network diagram show, from left to right, sagittal, coronal, and axial planes of the marmoset brain. DAN, dorsal attention network; DSN, dorsal somatosensory network; VSN, ventral somatomotor network; AN, auditory network; PVN, primary visual network; LVN, lateral visual network; DVN, dorsal visual network; BGN, basal ganglia network.

Nine RSNs were detected with group ICA in the awake condition: 2 higher-order cognitive networks, namely the DMN and DAN; 6 lower-order sensory networks, namely dorsal somatomotor network (DSN), ventral somatomotor network (VSN), auditory network (AN), primary visual network (PVN), lateral visual network (LVN), and dorsal visual network (DVN); and basal ganglia network (BGN). DVN was observed in 2 parts, left and right. All RSNs detected in each condition except for DMN are shown in Fig. 1. The results for DMN are shown in Fig. 4 and mentioned below.

Table 1 shows the RSNs detected under each sedative/anesthetic condition. Although DAN was detected separately in 2 components, in the Mida condition, most RSNs detected in the awake condition were preserved. In the Dex condition, all RSNs detected in the awake condition were observed. In the IsoDex condition, although almost all RSNs were detected, DVN was not detected and PVN and LVN were detected separately in 2 components. Detected RSNs in Propo condition were similar to IsoDex; DVN was not detected, and higher-order cognitive network was separated in 2 components. Almost all RSNs were not detected in the Alfa, Iso, and Sevo conditions. BGN was detected in all conditions except for Alfa condition. The RSNs among 7 groups tended to fall in 3 different subgroups that were (i) Mida and Dex, (ii) IsoDex and Propo, and (iii) Alfa, Iso, and Sevo.

**Table 1:** Resting-state networks detected with ICA in the awake condition.

	Awake	Mida	Dex	IsoDex	Propo	Alfa	Iso	Sevo
DMN	CP6	CP10	CP7	CP5	CP4, CP5			
DAN	CP12	CP18, CP21	CP19	CP9	CP10, CP12			
DSN	CP4	CP1	CP6	CP7	CP2, 3			
VSN	CP18	CP6	CP17	CP1	CP1			
AN	CP22	CP15	CP11	CP13	CP17			CP2
PVN	CP2	CP4	CP10	CP3, 21	CP11			
LVN	CP8	CP14	CP18	CP4, 6	CP7			
DVN	CP21, 23	CP22	CP12					
BGN	CP14	CP16	CP13	CP16	CP14		CP1	CP1

The components (CPs) indicate the number of components that each network was detected. When a cell contains several components, networks are detected by dividing them into more than two components. DAN, dorsal attention network; DSN, dorsal somatosensory network; VSN, ventral somatomotor network; AN, auditory network; PVN, primary visual network; LVN, lateral visual network; DVN, dorsal visual network; BGN, basal ganglia network.

**Fig. 2:**
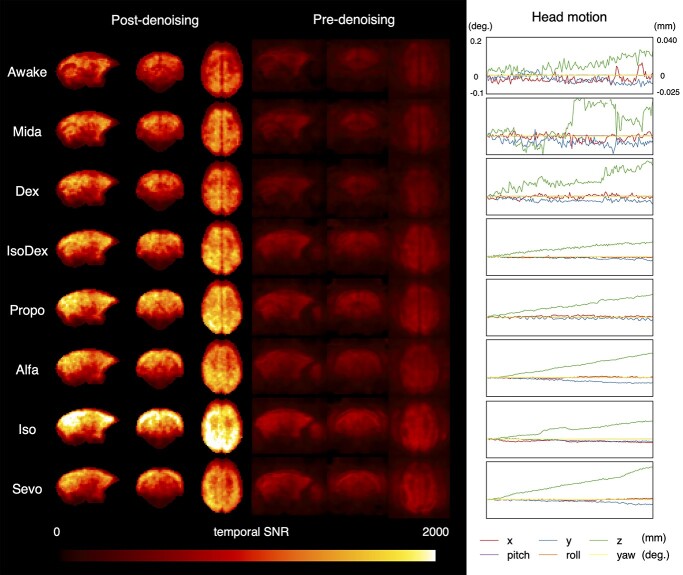
The temporal SNR and the head motion plot. The temporal SNR with the post-denoising data (left) or pre-denoising data (middle) was calculated with dividing mean value of signal by its standard deviation. The motion plot shows a scan-by-scan plot of the head motion in the 6 directions estimated by realignment: *x* (left/right), *y* (front/back), *z* (up/down), pitch (rotational direction of nodding and looking up), roll (rotational direction of moving the ear closer to the shoulder), and yaw (rotational direction of looking left/right). All data were calculated for each individual using 1 of the 12 sessions that were imaged and averaged across individuals.

Figure 2 shows temporal signal-to-noise ratio (SNR) calculated by dividing mean value of signal by its standard deviation and head motion plot estimated through re-alignment in data aquation. SNR tended to increase in anesthetic condition in both pre- and post-denoising data. For the doses used in this study, although Mida and Dex produced just light sedation, similar head motion with awake condition and almost no head movement was observed in IsoDex, Propo, Alfa, Iso, and Sevo.

### Evaluation of sedative/anesthetic effects on each region using graph theoretic analysis

**Fig. 3:**
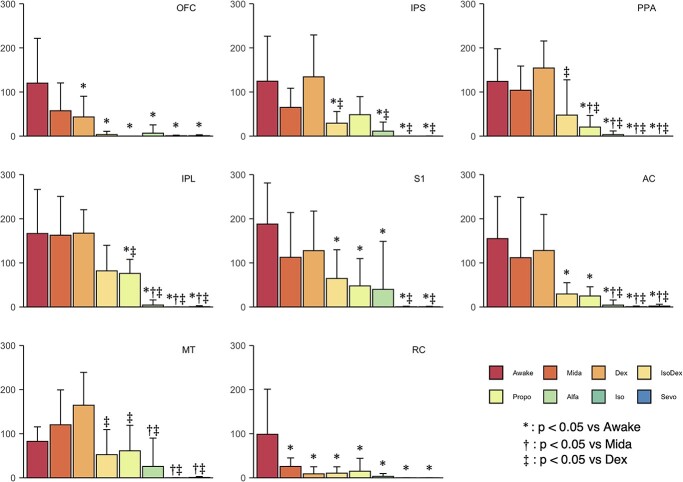
Betweenness centrality of the 8 regions determined to be significantly different between groups. The bar plots show betweenness centrality, calculated from each hemisphere in each individual, 8 hemispheres in total, in regions where statistically significant difference was observed with both one-way ANOVA and Tukey–Kramer test with *P* < 0.05. OFC, orbitofrontal cortex; IPS, intraparietal sulcus; PPA, postal parietal area; IPL, inferior parietal lobe; S1, primary somatosensory cortex; AC, auditory cortex; MT, middle temporal area; RC, retrosplenial cortex.

A graph theoretic analysis based on partial correlation coefficients was performed to estimate the region that causes the loss of RSNs due to the administration of sedatives or anesthetics, as found by ICA. In this study, regions with information transfer functions were defined as important regions for network formation, and the betweenness centrality was calculated to quantify how these information transfer functions are changed by sedatives or anesthetics. Brain region data were divided into 52 regions in the cerebral cortex of the hemisphere (Table 2) based on the common marmoset atlas data reported by Hashikawa et al. (2015). Figure 3 shows the betweenness centrality of the 8 regions determined to be significantly different between groups by one-way ANOVA and significantly changed among each condition by Tukey–Kramer test (the orbital frontal cortex, intraparietal sulcus, the postal parietal area, the inferior parietal lobe, the primary somatosensory cortex the auditory cortex, the temporopolar area, the middle temporal area, and the insular cortex). The orbital frontal cortex and the retrosplenial cortex were reduced in the almost sedate/anesthetic condition compared to awake condition, and there was no significant difference among sedate/anesthetic conditions. The alternation tendency of betweenness centrality among 7 groups tended to fall in 3 different subgroups, namely (i) Mida and Dex, (ii) IsoDex and Propo, and (iii) Alfa, Iso, and Sevo, as well as RSNs detected in ICA.

**Table 2:** Names and abbreviations of the 52 regions used in this study.

Lobe	Region	abb	Lobe	Region	abb
Frontal lobe	Olfactory bulb	OB	Occipital lobe	Primary visual cortex	V1
	Olfactory nucleus	ON		Secondary visual cortex	V2
	Frontal pole	FP		Third visual cortex	V3
	Orbital frontal cortex	OFC		Visual cortex area V6	V6
	Premotor cortex	PM	Limbic system	Anterior cingulate cortex	ACC
	Primary motor cortex	M1		Posterior cingulate cortex	PCC
	Dorsolateral prefrontal cortex	dlPFC		Retrosplenial cortex	RC
	Ventrolateral PFC	vlPFC		Subiculum	Subi
	Medial PFC	mPFC		Piriform cortex	PirC
	Ventromedial PFC	vmPFC		Parahippocampal gyrus	PhG
Parietal lobe	Intraparietal sulcus	IPS		Hippocampal formation	HF
	Postal parietal area	PPA		Amygdala	Amy
	Inferior parietal lobe	IPL		Bed nucleus of the stria terminalis	BNST
	Gustatory cortex	GC		Nucleus accumbens	AN
	Precuneus	Prec	Basal ganglia	Prostriate area	ProA
	Primary somatosensory cortex	S1		Claustrum	Cla
	Secondary somatosensory cortex	S2		Globus pallidus	GP
Temporal lobe	Auditory cortex	AC		Substantia nigra	SN
	Perirhinal cortex	PerC		Caudate nucleus	CaN
	Entorhinal cortex	EC		Putamen	Put
	Temporopolar area	TpA		Subthalamic nucleus	StN
	Middle temporal area	MT		Septal nucleus	SpN
	Inferior temporal area	IT		Medial geniculate nucleus	MGN
	Superior temporal rostral area	STRA		Thalamus	Tha
	Superior temporal polysensory cortex	STPC		Dorsolateral geniculate nucleus	dlGN
	Insular cortex	IC		Superior colliculus	SuC

The cerebral cortex of the hemisphere was divided into 52 regions in this study in based on the common marmoset atlas data reported by Hashikawa et al. (2015).

### Effects on higher-order cognitive networks

**Fig. 4:**
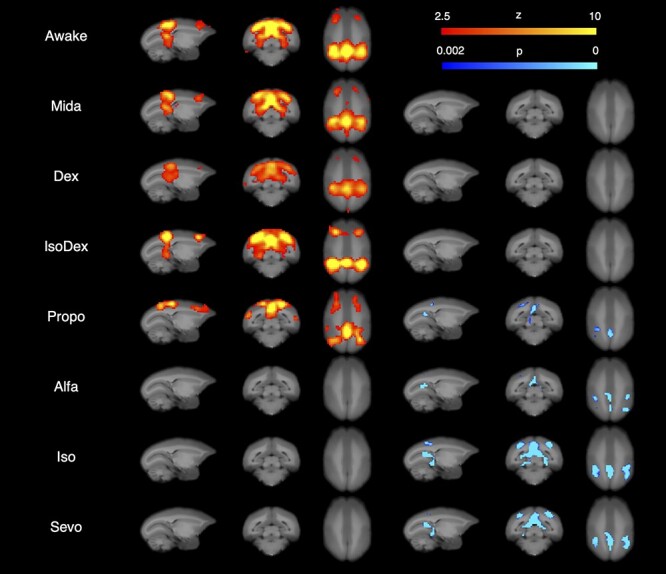
DMN detected in each condition and results of the dual regression analysis. The left column shows voxels with *z* values higher than 2.5, identified as red to yellow in ICA, and the right column shows voxels whose timecourses were significantly affected (*P* < 0.002) by sedatives or anesthetics in dual regression analysis, and these voxels are identified as blue to light blue.

Figure 4 shows results of ICA and dual regression analysis in DMN for each condition. Dual regression analysis extracted voxels whose time courses were significantly altered by sedatives/anesthetics, and Table 3 shows the regions where these voxels belonged and the numbers of these voxels. Premotor, intraparietal sulcus, postal parietal area, inferior parietal lobe, precuneus, posterior cingulate cortex, retrosplenial cortex, subiculum, the secondary visual cortex, the third visual cortex, the visual cortex area V6, and the prostriate area were extracted as the constructing regions of the DMN in the awake condition. In the Mida, Dex, and IsoDex conditions, DMN was detected with ICA, and there was no voxel whose timecourse was significantly changed compared with time courses in the awake condition. In the anesthetic conditions, except for Propo, the DMN was not detected, and some voxels were detected in the dual regression analysis. DMN could be observed only in the Propo condition among anesthetic conditions, but it differed from Mida, Dex, and IsoDex in that several regions were detected in dual regression analysis. The time courses for inferior parietal lobe and precuneus were significantly changed by administering Propo, Alfa, Iso, and Sevo; and Iso and Sevo inhalation changed the timecourses for almost all regions except for the premotor and postal parietal area. The number of voxels detected in the inhalational anesthetics with dual regression analysis tended to be higher than that of intravenous anesthetics, Propo and Alfa. Although the time courses in many regions were changed in the Alfa condition, the voxel count tended to be lower than that of inhalational anesthetics.

**Table 3:** Regions with statistically significant timecourse changes in DMN.

	Awake	Propo	Alfa	Iso	Sevo
PM	748				
IPS	4,122		222	1,710	1,673
PPA	3,062				
IPL	1,944	151	274	626	318
Prec	1,001	163	30	599	551
PCC	3,633	10	407	1,396	1,242
RC	1,974		363	562	470
V2	1,825		284	1,386	583
V3	3,775	7	591	1,197	1,205
V6	3,257	293	503	264	189
Subi	248			90	104
ProA	450			120	92
SuC	896		141	381	185

The number of voxels with a statistically significant change in the time course was detected by dual regression. The number in awake indicates the number of voxels with *z* > 2.5 in awake group-ICA. Mida, Dex, and IsoDex, for which no significant voxels were detected, are not shown. PM, premotor cortex; IPS, intraparietal sulcus; PPA, postal parietal area; IPL, inferior parietal lobe; Prec, precuneus; PCC, posterior cingulate cortex; RC, retrosplenial cortex; V2, secondary visual cortex; V3, third visual cortex; V6, visual cortex area V6; Subi, subiculum; ProA, prostriate area; SuC, superior colliculus.

**Fig. 5:**
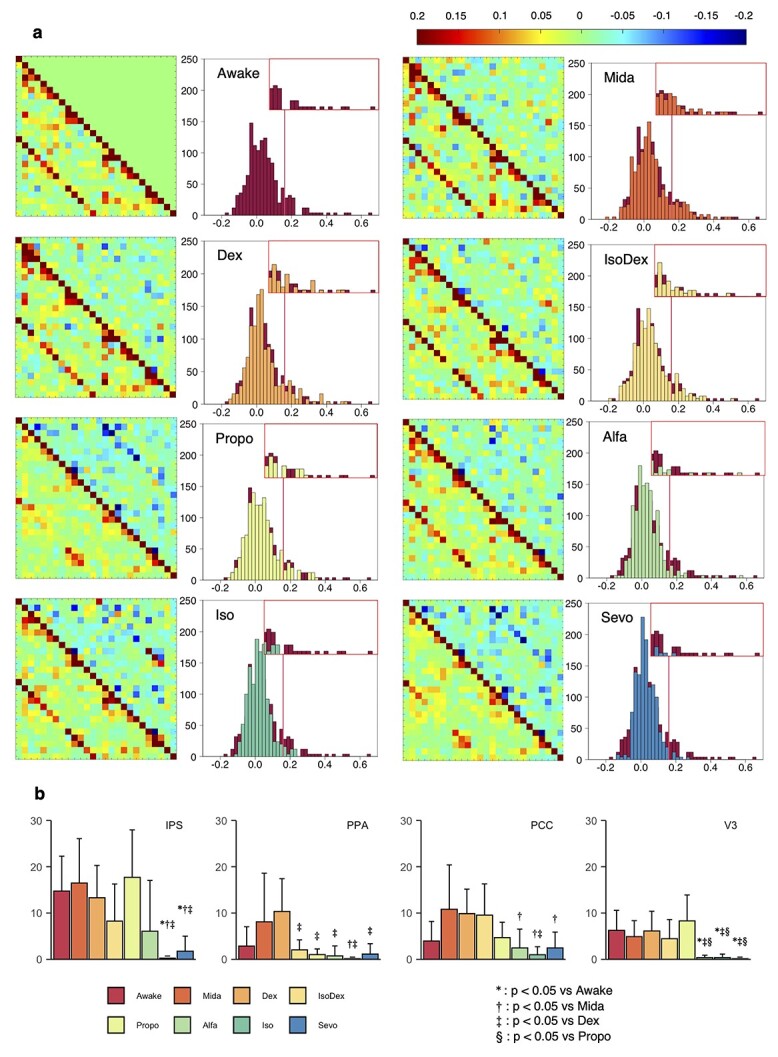
Partial correlate coefficients and betweenness centrality among DMN constituent regions. a) Partial correlate coefficients between the regions comprising the DMN, from left to right in the first row: awake, Mida, Dex, and IsoDex; from left to right in the second row: Propo, Alfa, Iso, and Sevo. The lower triangle of the matrix shows the calculated partial correlation coefficients, and the upper triangle shows the absolute difference from the awake condition. The upper left of the matrix shows the correlation within the left hemisphere, the lower right shows the correlation within the right hemisphere, and the others show the correlation between hemispheres. These matrices show the constituent areas of the DMN, respectively, from left or top, PM, IPS, PPA, IPL, Prec, PCC, RC, V2, V3, V6, Subi, ProA, and SuC in left and right hemispheres. The histogram shows the distribution of partial correlation coefficients, and the box located at the upper right of the histgram  shows the expanded distribution of partial correlation coefficients that are higher than the threshold calculated for the awake state. b) Betweenness centrality calculated among regions comprising the DMN. Only the regions where statistically significant difference was observed with both one-way ANOVA and Tukey–Kramer test with *P* < 0.05 are shown. OFC, orbitofrontal cortex; IPS, intraparietal sulcus; PPA, postal parietal area; PCC, posterior cingulate cortex; V3, third visual cortex.

Figure 5 shows the partial correlate coefficients and betweenness centrality among the regions constructing the DMN. Although the partial correlate coefficients tended to decrease under all sedative/anesthetic conditions, the degree of suppression was milder in Mida, Dex, and IsoDex compared with the anesthetic condition. The distribution of the partial correlation coefficients tended to be narrow and the shape of histogram tended to be sharp especially in Alfa, Iso, and Sevo (Fig. 5a). Under anesthetic conditions, except for Propo, frontal connectivity was particularly suppressed, as well as within the visual cortex. Figure 5b shows the results of the partial correlate coefficient-based graph theoretic analysis. The intraparietal sulcus, the postal parietal area, the posterior cingulate cortex, and the third visual cortex were shown to have significant differences among all conditions as information transfer site in the DMN defined with one-way ANOVA (Fig. 5b). Betweenness centrality of the intraparietal sulcus was significantly reduced in anesthetic condition with inhalational anesthesia. Although betweenness centrality of the postal parietal area and the posterior cingulate cortex in Mida and Dex were not significantly different from awake condition, they tended to increase. These alternations reflect the similar tendency of the number voxels as shown in Table 3. Regions with many voxels were detected in the dual regression analysis in Iso and Sevo condition such as the intraparietal sulcus and the posterior cingulate cortex; and the third visual cortex has lower betweenness centrality.

Supplementary Figures 1 and 2 show the results in DAN. The changes of network detection in ICA and the information transfer function shows similar tendency with DMN.

## Discussion

This study investigated the effects of sedatives/anesthetics on the resting-state network using ICA, dual regression analysis, and graph theoretic analysis to explore sedatives/anesthetics suitable for immobilization in higher-order brain function research using marmosets. As a result, it was shown that network formation was altered depending on the depth of sedation/anesthetics except for Propo, which showed unique effects.

### Resting-state networks observed in marmosets

Two higher-order cognitive networks, 6 lower-order sensory networks, and the BGN were detected in this study in awake condition. These networks have been reported in previous studies (Belcher et al. 2013; Ghahremani et al. 2016; Hori et al. 2020), and almost all of the reported networks were detected in this study. These results suggest that analysis in this study was performed with high quality. However, auditory network was detected instead of the saliency network in this study. Ghahremani et al. mentioned the possibility that the salience network can be identified as the auditory network. Containing similar regions to the salience network has been reported in previous studies, and the network detected in this study was judged as the auditory network because it has following features: the auditory cortex was major region constructing the network, the connectivity increased under sedative condition with Mida or Dex as lower-order sensory network, and the anterior cingulate cortex which is generally detected in the salience network was not detected in this study.

The visual cortex was detected as the region constructing the DMN in this study but it has not been reported in humans and macaques. The detected regions of visual cortex, V3a and V6a, are likely to have been detected as the constituent regions of the DMN in ICA because they are bordering the parietal regions and because V3a and V6a are adjacent to the MIP and LIP, which are the major constituent regions of the DMN in marmosets. Since the visual cortex in marmosets occupies a larger proportion of the brain than that in humans and macaques and is exposed on the surface (D’Souza et al. 2021), this may be a phenomenon unique to marmosets.

### Appropriate use of anesthetics in higher brain function analysis

Considering the appropriate use of sedatives and anesthetics in higher brain function research using marmoset rs-fMRI analysis based on the results of this study, light sedation using drugs with limited type of target receptors, such as Mida and Dex, combined with physical restraints, is thought to be the most feasible way to achieve both immobilization and network preservation. For the doses used in this study, although Mida and Dex produced light sedation but not deep sleep as in previous monkey studies (Miyabe et al. 2001), the combined use of the head post perfectly controlled body movement. Therefore, the use of mild restraints is recommended for sedation with Mida or Dex alone for fMRI imaging. Conversely, marmosets are smaller and weaker than macaques, so mild sedation and physical restraint are sufficient for immobilization. The sedative’s anxiolytic effect is expected to reduce psychological distress, and its use is recommended for animal welfare. In this study, there was no difference in effect between Mida and Dex: Mida directly inhibits the transmission of information between neurons in the cortex, while Dex does so indirectly through the locus coeruleus. This may explain the similar results between Mida and Dex, although the target receptors are different.

Sedation with Propo or IsoDex is recommended to achieve immobilization without restraints. In this study, no premedication was administered except for atropine, which prevents intratracheal secretion and vagal reflex, to eliminate the effect of premedication on neural activity. Consequently, the depth of sedation and anesthesia obtained was lower than that in a previous report (Autio et al. 2020; Hayashi et al. 2021), and endotracheal intubation could not be obtained with the protocol of co-administration of Iso and Dex. Therefore, premedication’s effect on sedative/anesthetic depth is significant, and the effect of premedication must be considered when measuring brain function with immobilization by sedation or anesthesia. However, co-administration of Iso and Dex resulted in a higher sedation depth than that associated with Dex alone; thus, more reliable immobilization can be expected. However, considering that Propo, which allowed endotracheal intubation, and the co-administration of Iso and Dex, which did not, were equally conservative, a reduced Propo dose may preserve the networks and reduce movement.

In the past, when sedatives/anesthetics were used for immobilization, the target was often set at a sedation or anesthesia depth at which resistance to stimuli applied during measurement could be acquired. However, in this study, endotracheal intubation was applied as an index of anesthesia depth, and the network’s preservation differed depending on the type of anesthetic. Our results indicate that the preservation of brain function is not equal even if the presence or absence of body movement to nociceptive stimuli is used as an index. Therefore, if anesthetics are selected based on the presence or absence of responses to stimuli in brain function studies, it is likely that the network’s preservation will differ depending on the anesthetic, which will significantly impact the results. On the other hand, it is a common finding that administering analgesics and opioids reduces the anesthetic required to maintain general anesthesia by reducing nociceptive input. Therefore, by conducting similar studies with different combinations of these non-sedating analgesics in addition to the dose of anesthetics, it may be possible to find a protocol that preserves more brain functions.

### Effects of sedatives/anesthetics on RSNs

The ICA analysis was performed to examine the effects of the immobilization protocol employed in this study on the RSN measurements. Initially, it was expected that the detected RSNs would be different due to depth of sedation or anesthesia and preserved the most in IsoDex condition. However, although RSNs were detected as expected in Mida, Dex, Alfa, Iso, and Sevo condition, those detected in Propo and IsoDex were different to expectations. More RSNs than expected were detected in Propo, and fewer RSNs in IsoDex (Grandjean et al. 2014; Paasonen et al. 2018). The higher-order cognitive networks lost beforehand the lower-order sensory network, and basal ganglia was last in almost all conditions. The thalamus is the main target region of sedatives and anesthetics and is important not only for maintaining wakefulness but also for the formation of a large-scale network of the brain. It has been reported that the connection of regions as distant as the frontal and occipital regions requires thalamic control of the firing rhythm (Reimann and Niendorf 2020). Therefore, it is expected that higher-order cognitive networks composed of distant regions are more susceptible to the effects of sedatives and anesthetics and that local networks or networks composed of regions close to the brainstem are more preserved. Furthermore, networks composed of local or brainstem regions that do not require thalamic intervention have stronger connectivity and may require more receptor-mediated inhibition.

A graph theoretic analysis was performed based on partial correlation coefficients calculated by dividing the network into 52 regions per a hemisphere in order to infer which regions of the network changed in function. Although the results show that the information transfer function was altered with a tendency similar to that of the ICA results in many regions, there are several regions where the function almost lost in all the sedate/anesthetic conditions, which are the orbitofrontal cortex and the retrosplenial cortex. The orbitofrontal cortex and the retrosplenial cortex were reduced in the almost sedate/anesthetic condition when compared to awake condition, and there was no significant difference among sedate/anesthetic conditions. Disrupting the activity of the orbitofrontal cortex severely impairs higher order cognitive and executive functions (Dalley et al. 2004; Eichenbaum 2017), and the retrosplenial cortex was thought to have a role in creating consciousness (Vogt and Laureys 2005). The information transfer function alternation in these regions in all conditions may reflect disruption of the higher-order cognitive function and the loss of consciousness. On the other hand, changes in information transfer function in the other regions showed similar trends between Mida and Dex, IsoDex and Propo, Alfa and inhalational anesthetics as in ICA analysis. The conservation of function in these regions is consistent with network conservation in ICA, suggesting that changes in RSN in sedatives and anesthetics are determined by the conservation of function in regions of high information exchange function such as the intraparietal sulcus, the inferior parietal lobe, and the auditory cortex. On the other hand, the functions of regions such as the orbitofrontal cortex and the retrosplenial cortex may have been expressed in a different way from the formation of RSN, such as loss of consciousness and loss of higher cognitive functions. Alfa, Iso, and Sevo suppressed neural activity to the extent that the information transfer function was hardly retained, resulting in the loss of lower-order RSNs and deep sedation. In the co-administration of IsoDex and Propo, where the residual information transfer function was moderate, the higher-order networks were lost while the lower-order sensory networks remained.

### Effects on higher-order cognitive networks

In addition, a detailed evaluation of the higher-cognitive network, which is often the focus of attention in higher brain functions, was conducted by a dual regression analysis. The results showed no statistically significant alternation of synchronicity under Mida, Dex, and the co-administration of Iso and Dex, while Propo, Alfa, Iso, and Sevo had decreased synchrony mainly in the temporal and parietal lobes. The postal parietal area, the posterior cingulate cortex, the precuneus, and the retrosplenial cortex have been reported as constitutive regions of DMN. The intraparietal sulcus and the inferior parietal lobe are not involved in DMN in humans; however, they were detected as constitutive regions in almost all previous reports of DMN in marmosets (Belcher et al. 2013; Liu et al. 2019); therefore, the intraparietal sulcus may be strongly involved as a constitutive DMN region in marmosets.

The results of graph theoretic analysis showed a similar tendency to that of dual regression analysis, and this could be considered a multifaceted evaluation of the effect of the sedatives/anesthetics on DMN of marmosets. Similar effects were observed between Mida and Dex and among Alfa, Iso, and Sevo. Initially, it was expected that the effects of Propo were similar to the other anesthetics because the imaging was carried out with aligned anesthetic depth; however, the effects of Propo were moderate compared to the other anesthetics. In Mida and Dex, the information transfer function was increased in several regions. The connectivity and the hub function of the posterior cingulate cortex could be increased under sedate condition with dexmedetomidine (Kim et al. 2017). Considering that there were regions with reduced function under all conditions in this study such as the retrosplenial cortex, and these regions were important for the level of consciousness and the higher-order cognitive functions, and that the higher-order cognitive networks were detectable with ICA despite their reduced function; the increased function in some regions may have been compensatory for the maintenance of higher-order brain function. From this, it can be said that when measuring higher-order networks under sedative/anesthetic conditions, sedatives that have little effect on the network, such as Mida and Dex, are most sensitive to detection. However, it should be noted that even if the network is maintained, there are areas of significantly altered activity in Mida and Dex. This is the first study to report topological network changes in nonhuman primates.

### Considerations for each sedative/anesthetic

The effects of each sedative/anesthetic on RSNs are discussed in the following sections.

1. ***Mida*** Mida binds to the benzodiazepine binding site of Gamma-aminobutyric acid (GABA)a receptors and enhances intracellular Cl^−^ ion influx, thereby inhibiting the generation of neuronal action potentials. In humans and mice, GABAa receptor agonists reduce feedback from the frontal cortex to the parietal and occipital cortices in the cerebral cortex (Boly et al. 2012; Lee et al. 2013a; Uhrig et al. 2014), which may be one of the mechanisms of the sedative anesthetic effect. In a study of the effects of Mida on the RSNs in humans, both higher-order cognitive networks and lower-order perceptual networks were detected under Mida treatment, but connectivity was enhanced in lower-order perceptual networks such as the DSN, VSN, and PVN, and attenuated in higher-order networks such as the DMN and DAN (Liang et al. 2015).In addition, it has been reported that administration of GABAa receptor agonists changes the topology of the network’s information transfer function in humans and rodents by anesthetic administration (Boly et al. 2012; Lee et al. 2013b; Jiang et al. 2015), and it can be inferred that the compensatory mechanism maintains the network even if partial connectivity or suppression of the information transfer function occurs. On the other hand, most studies that reported topological changes in information transfer function in humans and rodents used Propo or Iso. Although these anesthetics also primarily target GABAa receptors, their effects on NMDA receptors, AMPA, and kainate receptors are different from those of Mida (Reimann and Niendorf 2020); therefore, anesthetics may inhibit neuronal function more readily than Mida can. Therefore, Mida, which has fewer type of target receptors, may be superior in maintaining network and brain functions, but it is less effective in terms of immobilization, and complete immobilization may require mechanical restraint.
2. ***Dex*** The alpha2 receptor, the target receptor or Dex, is expressed on the nerve terminals of neurons in the locus coeruleus and inhibits postsynaptic firing by suppressing the release of the neurotransmitter noradrenaline from the nerve terminals. The locus coeruleus projects nerve fibers to the thalamus to promote and regulate neuronal firing, and since the thalamus acts on cortical neurons, it is thought to indirectly inhibit cortical function compared to GABAa receptor agonists by inhibiting the release of noradrenaline released by the locus coeruleus (Reimann and Niendorf 2020). The RSNs detected in the sedated condition by Dex were similar to those of Mida, suggesting that Dex had similar effects on the cerebral cortex even though the type of target receptors was different. However, Dex produces sedation closer to spontaneous sleep than that produced by GABA receptor agonists. Although there was no significant difference between Mida and Dex in this study because the measurements were made with equivalent light sedation, further increases in GABAergic receptor dosage may produce different results.
3. ***Propo, Alfa, Iso, and Sevo*** Propo, Alfa, Iso, and Sevo are anesthetics, unlike sedatives like Mida and Dex. Although Propo and Alfa were expected to produce similar results because their primary target receptors are GABAa receptors, the networks detected with Alfa were similar to those of inhalational anesthetics Iso and Sevo, showing disruption of higher-order networks associated with the frontal lobe and sensory networks. Inhaled anesthetics have been found to act on various receptors (Alkire et al. 2008; Reimann and Niendorf 2020) and cell membrane-bound ion channels to suppress the generation of action potentials (Pavel et al. 2020). Therefore, it is suggested that many neurons strongly suppressed not only the generation of the action potential by GABAa receptors but also the interneuronal transmission through various postsynaptic receptors, causing many networks to collapse unsustainably.Neurosteroid anesthetics, to which Alfa belongs, reportedly target many receptors (Tsutsui and Haraguchi 2016). Although GABAa receptors are considered their main target, other receptors include the *N*-methyl-d-aspartic acid, kainate, α-amino-3-hydroxy-5-methyl-4-isoxazolepropionate acid, σ, glycine, serotonin, nicotinic, and muscarinic receptors. The number of these receptors affected by neurosteroids is larger than that of Propo and is similar to inhalational anesthetics, suggesting that Alfa is more similar to inhalational anesthetics than Propo is.Propo caused a loss of frontal networks, several sensory networks, and temporal networks, but many sensory networks remained, such as the regions of significantly reduced synchrony, and also the higher-order networks, DMN and DAN. In human studies, Propo has been shown to reduce the frontal connectivity of higher-order networks during the loss of consciousness, but many networks measured in the awake state were detectable (Guldenmund et al. 2016). Propo reportedly has a weaker effect on receptors other than GABAa receptors and ion channels than that of inhalational anesthetics and Alfa. Therefore, although Propo produces a loss of consciousness and resistance to invasive stimuli by acting on multiple receptors, some sensory networks remain, and sensory-related information processing is expected to continue during general anesthesia. On the other hand, because Propo has more receptors and ion channels than Mida does, which also targets the GABAa receptors, it may have produced a deeper level of sedation and network loss than that produced by Mida.
4. ***Co-administration of Iso and Dex*** Tracheal intubation could not be achieved with the sedation protocol in this study. Although the detected networks and betweenness centrality were similar to those of Propo, the synchronicity among the regions constituting DMN was not statistically significantly altered in only IsoDex, which was capable of endotracheal intubation. It has been suggested in rodent studies (Grandjean et al. 2014; Bukhari et al. 2017) that the co-administration of multiple sedatives/anesthetics can minimize the effects on brain function by reducing the dose of each sedative/anesthetic, but our results contradict this. Considering the small effect of Dex alone on the networks and the small dose of Iso, the effect of drug interaction is significant, and if sedative depth is increased by combining multiple drugs, RSNs are expected to be affected even if the amount of Iso is small, and it is reasonable to assume that the network was preserved due to the low level of sedation in this case.In this study, only one protocol for co-administration was tested; therefore, it is necessary to test the protocol with different doses and drugs in the future. Propo is more conserved for information transfer function, and it may not be possible to say that co-administration is generally better at evaluating RSN. Autio et al. reported that endotracheal intubation was possible using ketamine in macaques (Autio et al. 2020), referred to as the dose of Iso and Dex in this study. Therefore, it is necessary to consider the preadministration effects when assessing brain function.

## Conclusion

RSNs became undetectable depending on the degree of sedation/anesthesia except for propofol. Propo showed unique effects unlike the other anesthetics even though it is classified in anesthetics and tended to preserve the network more efficiently than inhalational anesthetics do, even at the same depth of anesthesia that allowed endotracheal intubation. When measuring higher brain function in marmosets under sedation/anesthesia, sedatives or anesthetics should be selected in conjunction with physical immobilization, suggesting that maintaining general anesthesia with inhalation anesthetics is not recommended for preserving brain function.

## Data availability

The data used in this study have also used in other study. We plan to release the data as soon as all related studies are published.

## Funding

This work was supported by the program for Brain Mapping by Integrated Neurotechnologies for Disease Studies from the Japan Agency for Medical Research and Development (Grant Number JP21dm0207001 to HO), Japan Society for the Promotion of Science (Grant Number JP20H03630 to JH), and “MRI platform” as a program of Project for Promoting Public Utilization of Advanced Research Infrastructure of the Ministry of Education, Culture, Sports, Science and Technology, Japan (Grant Number JPMXS0450400622).

*Conflicts of interest statement*: There were no conflicts of interest for any of the authors involved in this study.

## Supplementary Material

Supplementary_bhac406
