# Supplementary material for: Effect of sedatives or anesthetics on the measurement of resting brain function in common marmosets
Source: Cereb Cortex. 2022 Oct 12;33(9):5148–62. doi: 10.1093/cercor/bhac406 (PMC10151911; doi:10.1093/cercor/bhac406)
Supplement: Supplementary_bhac406 [file supplementary_bhac406.docx]

**Supplementary data**


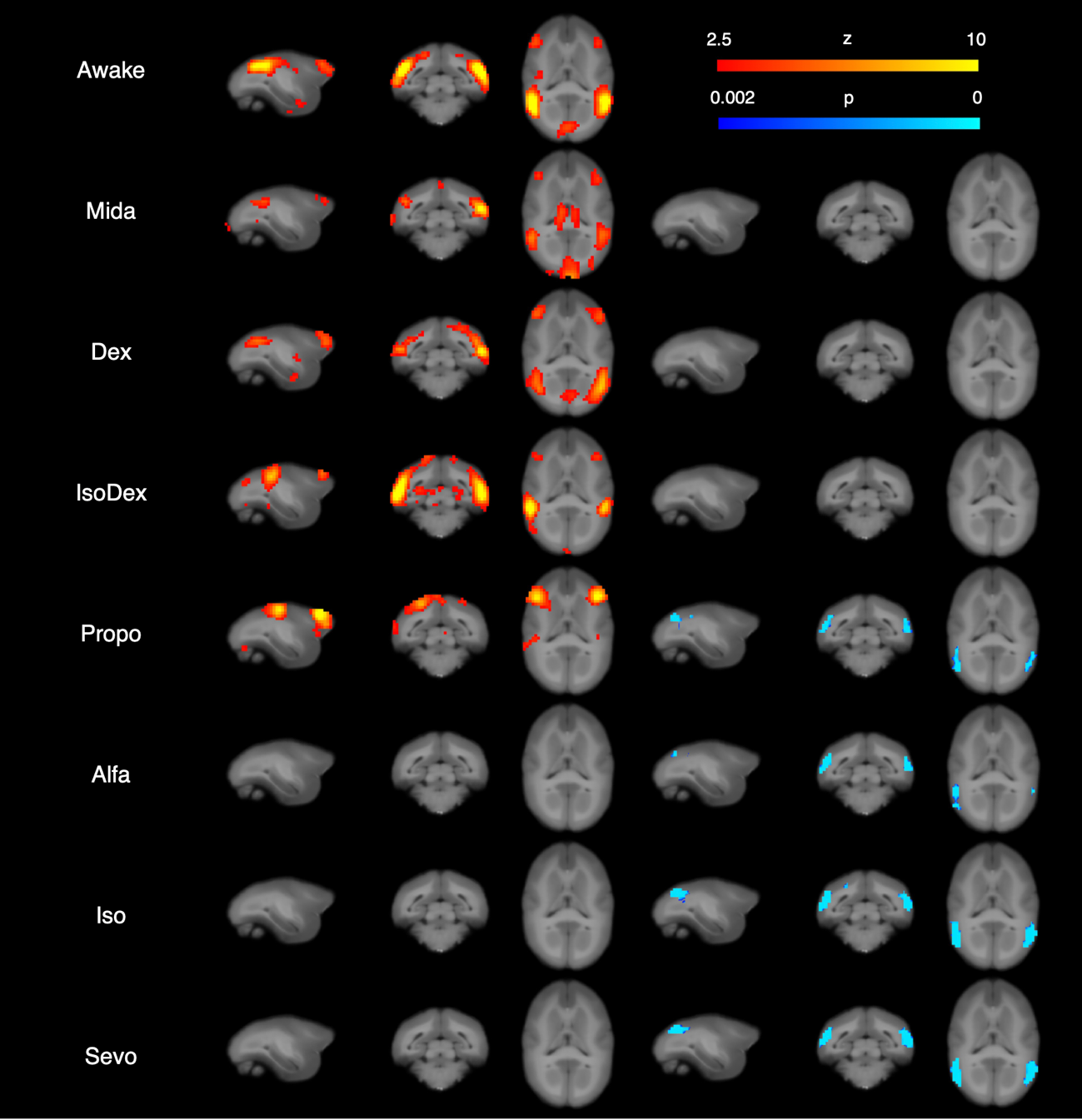


**Supplementary Figure 1.** DAN detected in each condition and results of the dual regression analysis.

The left column shows voxels with z values higher than 2.5, identified as red to yellow in ICA, and the right column shows voxels whose timecourses were significantly affected (p < 0.002) by sedatives or anesthetics in dual regression analysis, and these voxels are identified as blue to light blue.


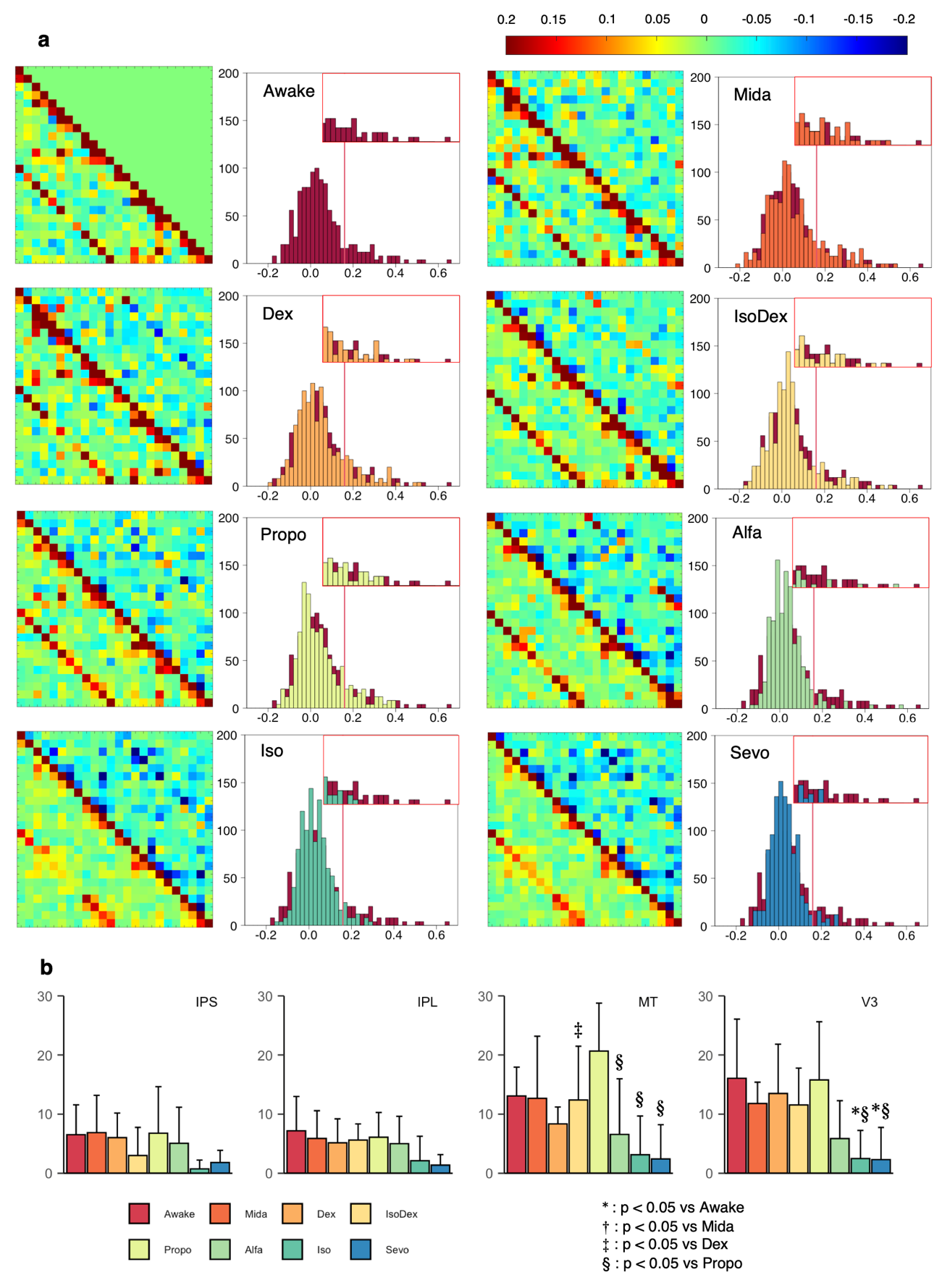


**Supplementary Figure 2.** Partial correlate coefficients and betweenness centrality among DAN constituent regions.

a: Partial correlate coefficients between the regions comprising the DAN, from left to right in the first row: awake, Mida, Dex, and IsoDex; from left to right in the second row: Propo, Alfa, Iso, and Sevo. The lower triangle of the matrix shows the calculated partial correlation coefficients, and the upper triangle shows the absolute difference from the Awake condition. The upper left of the matrix shows the correlation within the left hemisphere, the lower right shows the correlation within the right hemisphere, and the others show the correlation between hemispheres. These matrices show the constituent areas of the DAN, respectively, from left or top, PM, dlPFC, IPS, PPA, IPL, MT, IT, STPC, V1, V2, V3, and V6 in left and right hemisphere. The histogram shows the distribution of partial correlation coefficients, and the red box shows the expanded distribution of partial correlation coefficients that are higher than the threshold calculated for the awake state. b: Betweenness centrality calculated among regions comprising the DAN. Only the regions where statistically significant difference was observed with both one-way ANOVA and Tukey-Kramer test with p < 0.05 are shown. Abbreviations are as follows; OFC; orbitofrontal cortex, IPS; intraparietal sulcus, IPL; inferior parietal lobe, MT; middle temporal area, V3; third visual cortex.

|  | Awake | Propo | Alfa | Iso | Sevo |
| --- | --- | --- | --- | --- | --- |
| PM | 647 |  |  |  |  |
| dlPFC | 516 |  |  |  |  |
| IPS | 1861 |  |  |  |  |
| PPA | 442 |  |  |  |  |
| IPL | 2321 |  | 21 | 83 |  |
| MT | 7937 | 1982 | 1294 | 4094 | 4115 |
| IT | 6121 | 2 | 47 | 378 | 103 |
| STPC | 814 |  |  |  |  |
| V1 | 3319 | 245 |  | 222 | 51 |
| V2 | 2075 | 121 | 224 | 617 | 191 |
| V3 | 3716 | 1043 | 77 | 1943 | 1714 |
| V6 | 2635 |  | 113 | 133 | 16 |

**Supplementary Table 1.** Regions with statistically significant timecourse changes in DAN.

The number of voxels with a statistically significant change in the time course was

detected by dual regression. The number in Awake indicates the number of voxels with z > 2.5 in Awake group-ICA. Mida, Dex, and IsoDex, for which no significant voxels were detected, are not shown. Abbreviations are as follows; PM; premorter cortex, dlPFC; dorsolateral prefrontal cortex, IPS; intraparietal sulcus, PPA; postal parietal area, IPL; inferior parietal lobe, MT; middle temporal area, IT; inferior temporal area, STPC; superior temporal polysensory cortex, V1; primary visual cortex, V2; secondary visual cortex, V3; third visual cortex, V6; visual cortex area V6.
